# Cellular and oxidative stress responses of *Mytilus galloprovincialis* to chlorpromazine: implications of an antipsychotic drug exposure study

**DOI:** 10.3389/fphys.2023.1267953

**Published:** 2023-09-13

**Authors:** Federica Impellitteri, Kateryna Yunko, Viktoria Martyniuk, Vira Khoma, Giuseppe Piccione, Oksana Stoliar, Caterina Faggio

**Affiliations:** ^1^ Department of Veterinary Sciences, University of Messina, Messina, Italy; ^2^ Ternopil Volodymyr Hnatiuk National Pedagogical University, Ternopil, Ukraine; ^3^ Ternopil Scientific Research Forensic Center of the Ministry of Internal Affairs of Ukraine, Ternopil, Ukraine; ^4^ Department of Chemical, Biological, Pharmaceutical and Environmental Sciences, University of Messina, Messina, Italy

**Keywords:** pharmaceuticals, bivalve mollusc, ecotoxicity, cell volume regulation, oxidative stress, antioxidants

## Abstract

**Introduction:** Bivalve molluscs like *Mytilus galloprovincialis* are valuable bioindicators due to their filter-feeding lifestyle, wide distribution, and ability to concentrate xenobiotics. Studying the effects of pharmaceuticals on these molluscs is crucial given their presence in surface waters. This study investigated the response of *M. galloprovincialis* to chlorpromazine (Cpz), an antipsychotic with antiviral activity against influenza, HIV, and coronaviruses in human cells.

**Methods:** In this study, we examined the 14-day impact of chlorpromazine (Cpz) on the model species *M. galloprovincialis* at two concentrations (Cpz 1: 12 ng ^L-1^ or 37 pM; Cpz 2: 12 µg ^L-1^ or 37 nM). To ensure controlled exposure, a stock solution of Cpz was prepared and introduced into the tanks to match the intended concentrations. Seawater and stock solutions were refreshed every 48 h. The primary focus of this study centered on evaluating cell viability, cell volume regulation, and oxidative stress indicators.

**Results:** Although cell volume regulation, as assessed by decreasing regulatory volume Regulation volume decrease, did not show statistically significant changes during the experiment, digestive cell viability, on the other hand, showed a significant decrease (*p* < 0.01) in the Cpz 2 group, suggesting effects on the general health and survival of these cells. Biochemically, in both Cpz 1 and Cpz 2, superoxide dismutase activity increased, while catalase (CAT) decreased, causing an elevated lipid peroxidation thiobarbituric acid-reactive substances and protein carbonyls, particularly in the Cpz 2 group. The level of reduced glutathione (GSH) increased in both exposures, whereas the level of GSSG increased only in the Cpz 1 group. Consequently, the GSH/GSSG ratio was elevated in the Cpz 2 group only.

**Discussion:** A comparison of the magnitudes of anti- and pro-oxidative manifestations indicated a pro-oxidative shift in both exposures. These findings show that Cpz induces non-specific symptoms of biochemical and cellular disturbances in *M. galloprovincialis* even at the low picomolar concentration.

## 1 Introduction

Bivalve molluscs like *Mytilus galloprovincialis* are valuable bioindicators due to their filter-feeding lifestyle, wide distribution, and ability to concentrate xenobiotics (Pain-Devin et al., 2014; Freitas et al., 2020; Pagano et al., 2020; Curpan et al., 2022). Particularly, their validity to reflect the impact of micropollutants, like pharmaceuticals, has been proven in recent years (Faggio et al., 2018; Piedade et al., 2020; Martyniuk et al., 2022a; Martyniuk et al., 2022b; Martyniuk et al., 2023; Chahouri et al., 2023; Impellitteri et al., 2023a; Impellitteri et al., 2023b). The pharmaceuticals represent a comparatively novel group of micropollutants of increasing concern given their widely discharged into water bodies on a continual basis (Bottoni et al., 2010; Fabbri and Franzellitti, 2016; Turani et al., 2019; Martyniuk et al., 2023; Porretti et al., 2022). They fall into the water along with the insufficiently purified sewage from water treatment plants and directly from households in rural areas, which often lack wastewater treatment facilities (Burgos-Aceves et al., 2018; Burgos-Aceves et al., 2021). Among the most expected pharmaceuticals of emerging concern that leaked into the surface waters, antipsychotic drugs attract special attention (Escudero et al., 2021; Moreira et al., 2022; Sehonova et al., 2017). They are increasingly used to treat a wide range of diseases. Currently, about 100 million people worldwide are affected by neurological disorders and represent 20% of the global burden of disease (Aleya and Uddin, 2020). Antipsychotics have a complex pharmacological profile, acting on multiple receptors common for different phyla, which are often referred to as ‘dirty drugs’ (Escudero et al., 2021). Therefore, their effects as water contaminants are particularly complicated to indicate and predict. This statement is completely related to chlorpromazine (Cpz), a member of the phenothiazine family of drugs. Its discovery 70 years ago marked the beginning of the application of the heterogeneous class of antipsychotics in medicine. Cpz is listed in the World Health Organization (WHO) Model Lists of Essential Medicines lists as one of five major medicines used in psychotic disorders for curing schizophrenia and schizophrenia-like psychoses (Dudley et al., 2017). With respect to the assessment of schizophrenia as a serious mental illness affecting around 1% of the adult population worldwide, and recommended dosage of Cpz in the range being 400–800 mg/day, the potential input of this drug into surface water can be significant.

Data concerning the concentrations of Cpz in surface waters are scarce. In the influents of municipal wastewater treatment plants in Beijing, China, the concentration of Cpz reached levels of 5–364 ng L^−1^ (Yuan et al., 2013). However, some local water sources were reported to contain much higher Cpz concentrations. For example, Cpz was found among 15 particularly hazardous chemicals present at high concentrations (mg L^−1^) in hospital wastewater (Frédéric and Yves, 2014). The persistence of Cpz in river water and strong adsorption on sediments have also been reported (Jiménez et al., 2016). Moreover, its presence in the water is expected to increase due to the development of new directions of Cpz applications in medicine. Its interference with topoisomerase action, similar to several anti-cancer drugs, has attracted attention to its potential as an anti-cancer agent (Darkin et al., 1984), and recent studies have confirmed its impact on the cell cycle in the oncogenesis (Lee et al., 2015; Xu et al., 2022). Another novel direction for Cpz utilization in medicine is associated with its antiviral activity against SARS-CoV-2 achieved through a membrane destabilizing effect (Stip et al., 2020).

These prospects increase the likelihood of Cpz entering surface waters and underscore the urgency of understanding its effects on aquatic habitats. The current body of research in this area is still limited, contradictory, and primarily focused on physiological indices. A high toxic pressure of Cpz has been confirmed in fish plasma models, with a concentration of 36 ng L⁻^1^ corresponding to critical environmental concentrations (CEC) (Sanderson et al., 2004). In the larvae of *Mytilus galloprovincialis*, Cpz inhibited metamorphosis by 50% (IC₅₀) at a concentration of 1.6 × 10⁻⁶ M (Yang et al., 2011). Nonetheless, in the rotifer *Brachionus calyciflorus*, known for its higher sensitivity to drugs compared to other invertebrates, exposure to Cpz significantly decreased life expectancy at hatching, the net reproduction rate, generation time, population growth rate, and dopamine concentration. This effect was observed at relatively high concentrations of 0.8, 1.2, 1.6, 2.0, 2.4, 2.8, and 3.2 mg/L (in acute exposure), as well as at concentrations of 0.125, 0.25, and 0.5 mg/L over a 7-day period (Feng et al., 2022). Biochemical responses to Cpz, particularly oxidative stress manifestations, are reported mainly in fish and in acute exposures to high (hundreds of µg and mg per L) concentrations (Li et al., 2008; Atama et al., 2022). To the best of our knowledge, biochemical responses of bivalve molluscs to Cpz in low (56 nM) concentrations were studied only in our previous works (Khoma et al., 2020; Khoma et al., 2022).

Based on this limited information concerning the effects of Cpz on aquatic animals and its complex pharmacological profile (Escudero et al., 2021), we expected that the common manifestations of cellular and biochemical stress responses can reflect the severity of the Cpz impact. Thus, the focus of this study was to indicate the effect of the Cpz on the marine mussel *Mytilus galloprovincialis* in the sub-chronic exposure. To reflect the effect of Cpz, we studied cell viability, cell volume regulation, and oxidative stress indexes. Among the markers of exposure, we selected the oxidative stress response because prolonged or high-dose treatment with pharmaceuticals causes as a side effect reactive oxygen species (ROS) generation. This is confirmed by a large body of data concerning the activities of antioxidant enzymes in exposed marine species (see the review of Fabbri and Franzellitti, 2016). For this study, we selected the first-line defense antioxidant enzymes superoxide dismutase (SOD) and catalase, and non-enzymatic antioxidant and most abundant low-weight intracellular thiol glutathione that directly reacts with ROS and other reactive species, and most approved indexes of the oxidative injury of lipids and proteins (Lushchak, 2012; Ighodaro and Akinloye, 2017; Lushchak and Storey, 2021). Two concentrations of Cpz were selected for this study. The low concentration corresponded to the level detected in sewage treatment plant effluents (Baresel et al., 2015) and fell within the limits reported for surface waters (Yuan et al., 2013). This concentration was also compared with the critical environmental concentration estimated in the freshwater invertebrate *Gammarus pulex* and the human therapeutic plasma concentration of 36 ng L⁻^1^ (Miller et al., 2019). The high concentration corresponded to levels approximately equal to 1/100 of the EC50 value (1.805 mg L⁻^1^) for *Daphnia magna* (Oliveira et al., 2015). Additionally, it was equivalent to the concentration of Cpz applied in our previous study to the freshwater bivalve mollusc *Unio tumidus* (18.0 μg L⁻^1^ for 14 days), which induced oxidative stress in this species (Khoma et al., 2020; Khoma et al., 2022).

## 2 Materials and methods

### 2.1 Experimental design

During the experiment, 200 bivalve molluscs of *Mytilus galloprovincialis* were collected and purchased from a commercial farm at “Faro Lake” by the “FARAU SRL Company, Frutti di Mare” in Messina, Italy. The specimens were divided into four groups (two replicates of 25 animals per group) and acclimatised for about 2 weeks in eight aquariums with 25 L of water each, equipped with oxygen aerators. Prior to commencing the main study, preliminary stability tests with Cpz were conducted in our experimental conditions. These tests involved analyzing the Cpz concentration at regular intervals over a period of 2 days within the tanks, ensuring that any potential degradation or decline was carefully monitored. Two Cpz concentrations (Cpz 1: 12 ng L^-1^; Cpz 2: 12 μg L^-1^) were administered to mussels for 14 days. Untreated molluscs (C-group) were also examined after the same period. Every 2 days, the tank’s water was changed, and the chemicals were also prepared and refilled at the same time. The molluscs were fed with filtered lake water that had been enriched with nutrients and was given by the same company that provided animals during the experiment (salinity: 3.4% ± 0.02%, pH: 7.6 ± 0.01, T: 16.77°C ± 0.09°C). The experimental room had a light mode of 12:12 light-dark and was 18°C. After every water exchange, the salinity, pH, and temperature of the water were checked and matched the same standards as initially. After exposure, the molluscs were immediately anesthetized on ice and dissected.

### 2.2 Haemolymph collection

To perform cell viability tests on hemolymph, approximately 1 mL of hemolymph was extracted from the anterior adductor muscle of each mussel using a 1 mL plastic syringe equipped with a 23-gauge needle, following the established protocol described in the literature (Impellitteri et al., 2022; Pagano et al., 2022; Tresnakova et al., 2023b).

### 2.3 Cell viability of digestive gland cells and haemocytes

The present study investigated the effects of Cpz on cell viability by analyzing both haemolymph and digestive glands collected from pooled mussels (*Mytilus galloprovincialis*). The DG cells were isolated following a series of steps. Initially, the digestive glands were obtained from randomly selected animals in the aquarium. The glands were then mechanically minced and washed with a calcium and magnesium-free solution (CMFS; 1,100 mOsm; pH 7.3). Subsequently, the resulting mixture was transferred to a tube containing 6 mL of dissociating solution (0.01% collagenase) in CMSF and stirred slowly for 60 min at 18°C. The suspension was then filtered and centrifuged (500 rpm/10 min/4°C). After removing the supernatant, the cells were resuspended twice with saline. The samples were returned to the thermostatic bath at 18°C for another hour and then analysed using the staining techniques outlined below. Two distinct colorimetric assays, the Trypan Blue (TB) exclusion method and Neutral Red (NR) retention assay, were employed for this purpose. In the TB exclusion method, cells were stained with Trypan blue, which selectively enters non-viable cells with compromised membranes. Viable cells, in contrast, prevent the uptake of Trypan blue dye. The percentage of unstained cells in the cell suspension was used as an indicator of cell viability and helped assess the extent of cellular damage caused by Cpz. The neutral red retention assay (NR) was conducted to further assess lysosomal membrane stability. Following a 15-min incubation period, living cells exhibited the ability to absorb and bind the neutral red dye within their lysosomes. The extent of dye retention provided valuable insights into the stability of the lysosomal membrane, crucial for understanding the impact of Cpz on cellular structures. Cell viability was assessed according to the following formula:

$$cell\;viability\;\left(\%\right)=\frac{number\;of\;viable\;cells}{total\;number\;of\;cells}\times 100$$

This assay is based on the work of Torre et al. (2013) and has been widely used to study the effects of various substances on living cells, including recently (Porretti et al., 2023).

### 2.4 Regulation of volume decrease (RVD) evaluation

To evaluate the RVD assay in the digestive gland (DG) cells of *Mytilus galloprovincialis*, we adopted the methodology outlined by Pagano et al. (2016) and Impellitteri et al. (2023b). In particular, the procedures for isolating digestive gland cells and preparing samples for the RVD technique remain consistent with those outlined in section 2.3. Cell samples from DG were placed on a slide and observed under a light microscope (Carl Zeiss Axioskop 20, Wetzlar, Germany), connected to a Canon 550D camera. Three photos were taken sequentially after a gentle isotonic solution wash. Then, the sample was gently washed with a hypotonic solution (800 mOsm) before capturing images. The images were captured every minute for the first 10 minutes and then every 5 minutes for the subsequent 25 minutes. ImageJ software was used to compare the cell area of the exposed cells with that of the control group. In each experimental group, images were taken of 15 cells to ensure reliable measurements for the comparison.

### 2.5 Biomarkers of oxidative stress

For the biochemical analysis, the samples of digestive gland tissues in single-use aliquots were prepared individually from eight mussels in each experimental assay. Homogenates 10% w/v in 0.1 M phosphate buffer, pH 7.4, containing 100 mM KCl and 1 mM EDTA, as well as 0.1 mM phenylmethylsulfonyl fluoride (PMSF) for proteolysis inhibition were utilized. Applied assays are described in detail in the Supplements (Martyniuk et al., 2022a; Martyniuk et al., 2022b).

For the enzyme assays, homogenates were centrifuged at 6,000 x *g* for 10 min. The resulting supernatants were kept at −40°C for measurements. The protein concentration in the supernatant (soluble protein) was measured according to the method of Lowry et al. (1951), using bovine serum albumin as the protein standard. The absorbance values were measured on a spectrophotometer UV/Vis ULAB 102UV (China).

Superoxide dismutase (SOD, EC 1.15.1.1) activity was measured according to the non-enzymatic assay based on aerobic reduction of nitro-blue tetrazolium (NBT) in the presence of phenazine methosulphate and NADH (Fried, 1975). The reduction of NBT was registered at 560 nm. The results were expressed as SOD units per mg of soluble protein (one unit of SOD is defined as the amount of enzyme that causes 50% inhibition of NBT reduction).

Catalase (CAT, EC 1.11.1.6) activity was measured by monitoring the decomposition of H_2_O_2_ according to Aebi (1974). The reaction was measured at 240 nm (*ε* = 0.04 mM^-1^ cm^-1^) and expressed as μmol min^-1^·mg^-1^ soluble protein.

Total glutathione (reduced plus oxidized, GSH plus GSSG, correspondingly) concentration was quantified in the protein-free extract of 10% w/v homogenate by the glutathione reductase recycling assay (Griffith, 1980) using 5,5′-dithio-bis(2-nitrobenzoic acid) DTNB for thiols quantification. To obtain the extract, 20% sulfosalicylic acid was added to homogenate in the proportion 1:3 and mix was centrifuged (12,000×*g*, 4 °C). Standards were prepared from GSH, and concentrations were expressed as µmol per g wet weight. To estimate the GSSG (in GSH-Eq) level, the protein free sample was treated with 2-vinylpyridine prior to the assay. The concentration of GSH was calculated as the difference between the total glutathione and GSSG concentrations. The redox-index of glutathione (RI GSH) as the ratio of concentrations GSH/GSSG was calculated.

The products of lipid peroxidation (LPO) were determined in the 10% w/v homogenate as the production of thiobarbituric acid-reactive substances (TBARS) after the sedimentation of proteins in sulfosalicylic acid (Ohkawa et al., 1979). The absorbance of the chromogen was determined at 532 nm (*ε* = 1.56 × 10^5^ M^-1^ cm ^-1^). Concentrations were expressed as nmol·g^-1^ fresh weight (FW).

Protein carbonyls (PC) as an index of protein oxidation were analyzed in the sediments of proteins from digestive gland tissue in sulfosalicylic acid with 2.4-dinitrophenylhydrazine (DNPH) (Reznick and Packer, 1994). PC concentrations were calculated from the absorbance at 370 nm using a molar absorption coefficient of 22.000 M^-1^ cm^-1^ and expressed as µmol PC·g^-1^ FW.

### 2.6 Statistical analysis

The one-way ANOVA and Tukey’s *post hoc* test were used to compare the viability of digestive gland and haemocyte cells. Results for RVD tests were obtained by employing one-way ANOVA and Tukey’s *post hoc* multiple comparison tests. The significance of the *p*-value was established as *p* < 0.05. The results were expressed as mean ± standard error. For all biochemical traits, the sample size was six from six individuals. Data were tested for normality and homogeneity of variance by using the Shapiro-Wilk test and Levene’s tests, respectively. Whenever possible, data were normalized by Box-Cox common transforming method. One-way ANOVA was used to test the effect of treatments, followed by *post hoc* procedures. Pearson correlation analysis was performed to analyze the strength and direction of the linear relationship between two continuous variables. The correlation was significant at *p* < 0.05 level. For the data that were not normally distributed, non-parametric tests (Kruskall–Wallis ANOVA and Mann–Whitney U-test) were performed. Normalized, Box-Cox transformed data were subjected to the principal component analysis (PCA) to assess the relations between measured parameters utilizing the rotation method Varimax with Kaiser Normalization, and Canonical discriminant analysis was utilized for the separation of the exposed groups. The IBM SPSS Statistics version 24 software for Windows was used for calculations.

The calculation of the balance between the levels of antioxidants and prooxidative manifestations (an index of Antioxidant/Pro-oxidant Balance, APB) was accomplished. It was defined as the shift of the equilibrium between antioxidant (SOD, CAT, GSH) and pro-oxidative manifestation (TBARS, Protein carbonyls, GSSG) values. Mean values in each group were utilized for this calculation and marked as Mi for exposed groups and Mc for control group. Each index in the exposed groups was standardized as a rate of deviation from control value A = 100*(Mi-Mc)/Mc. The integrative index APB was calculated as the ratio (SOD+CAT+GSH)/(TBARS+PC+GSSG) in the relative units assuming that the mean value of APB in the control group equalled 1.0 (Khoma et al., 2020).

## 3 Results

### 3.1 Cell viability of digestive gland cells and haemocytes

After 14 days of exposure, the viability of digestive gland cells remained high across all conditions tested, with a percentage of cells found alive above 90%. However, significant differences were observed between the groups (Table 1). The group exposed to the higher concentration (Cpz 2) exhibited significantly lower viability (”**” *p* < 0.01) compared to the control group (C) and to the lower concentration (Cpz 1; “aa” *p* < 0.01). This observation was consistent in both the NR and TB tests.

**TABLE 1:** Percentage of the digestive cells’ viability in *Mytilus galloprovincialis* after 14 days of exposure to chlorpromazine (Cpz). The tests conducted were Trypan Blue (TB) exclusion method and Neutral Red (NR) retention assay. Values are presented as mean ± SE (n = 12).

Method	Sampling time	Tested group		
		Control (0 mg/L)	Cpz 1 (12 ng L^-1^)	Cpz 2 (12 μg L^-1^)
TB	14 Days	99% ± 1	98% ± 0.6	95% ± 2.2**aa
NR	14 Days	99% ± 0.1	98% ± 0.2	94% ± 0.7**aa

One-way ANOVA, was used to assert the difference between the control group and for comparing the treats to each other. The * represents the differences compared to the control group: *p < 0.05, **p < 0.01. The letter “a” represents the differences in the treated groups; Cpz 2 was compared to Cpz 1: “a” < 0.05, “aa” < 0.01.

On the other hand, following the same exposure period, hemolymph cells from all tested conditions, displayed high lysosomal membrane stability, with over 90% cell viability. This trend was consistent across the groups and was also reflected in the Trypan Blue exclusion method (Table 2).

**TABLE 2:** Percentage of the haemocytes’ viability in *Mytilus galloprovincialis* after 14 days of exposure to chlorpromazine (Cpz). The tests conducted were Trypan Blue (TB) exclusion method and Neutral Red (NR) retention assay. Values are presented as mean ± SE (n = 12).

Method	Sampling time	Tested group		
		Control (0 mg/L)	Cpz 1 (12 ng L)	Cpz 2 (12 μg L)
TB	14 Days	98% ± 0.5	98% ± 0.4	97% ± 0.6
NR	14 Days	98% ± 0.2	98% ± 0.3	96% ± 0.8

One-way ANOVA, was used to assert the difference between the control group and for comparing the treats to each other. The * represents the differences compared to the control group: *p < 0.05, **p < 0.01. The letter “a” represents the differences in the treated groups; Cpz 2 was compared to Cpz 1: “a” < 0.05, “aa” < 0.01.

### 3.2 Regulation of volume decrease (RVD) evaluation

*M. galloprovincialis* is an osmoconform organism, which translates into the ability of the cells of the digestive gland to regulate their volume in the presence of a hypotonic solution. This regulatory mechanism results in swelling and subsequent gradual restoration of their original volumetric dimensions over time. Consequently, under physiological conditions, when exposed to a hypotonic environment, digestive gland cells swell and then gradually return to their original volume, demonstrating their ability to maintain cellular homeostasis.

**FIGURE 1:**
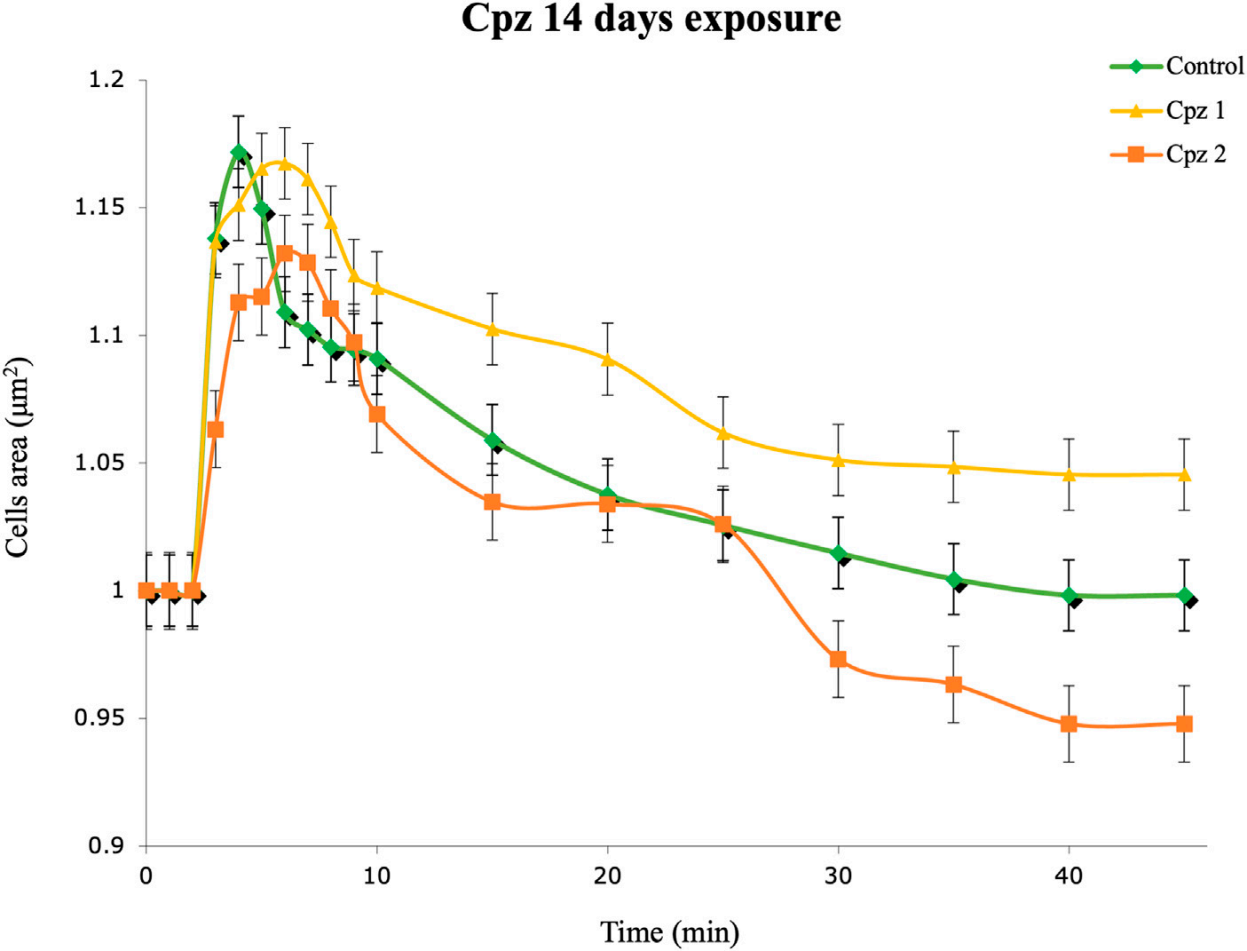
Regulation volume decrease (RVD) of digestive gland cells of *Mytilus galloprovincialis* after 14 days of exposure to Chlorpromazine (Cpz). Rhombuses (◆) represent control (0 mg L^−1^); triangles (▲) represent the Cpz 1 group (12 ng L^-1^of Cpz); squares (■) represent the Cpz 2 group (12 μg L^-1^of Cpz). Data are presented as mean ± SE (n = 12).

Our statistical analysis revealed no statistically significant differences in the results of the Cpz exposure experiments compared to the control group. However, upon closer examination of the data, subtle variations can still be observed between the tested and control groups. In the Cpz 1 group, we observed a trend whereby DG cells appeared to have difficulty returning to their initial volume, resulting in prolonged swelling (Figure 1). Furthermore, DG cells exposed to the highest concentration of Cpz showed unusual contraction patterns.

### 3.3 Oxidative stress indexes

**FIGURE 2:**
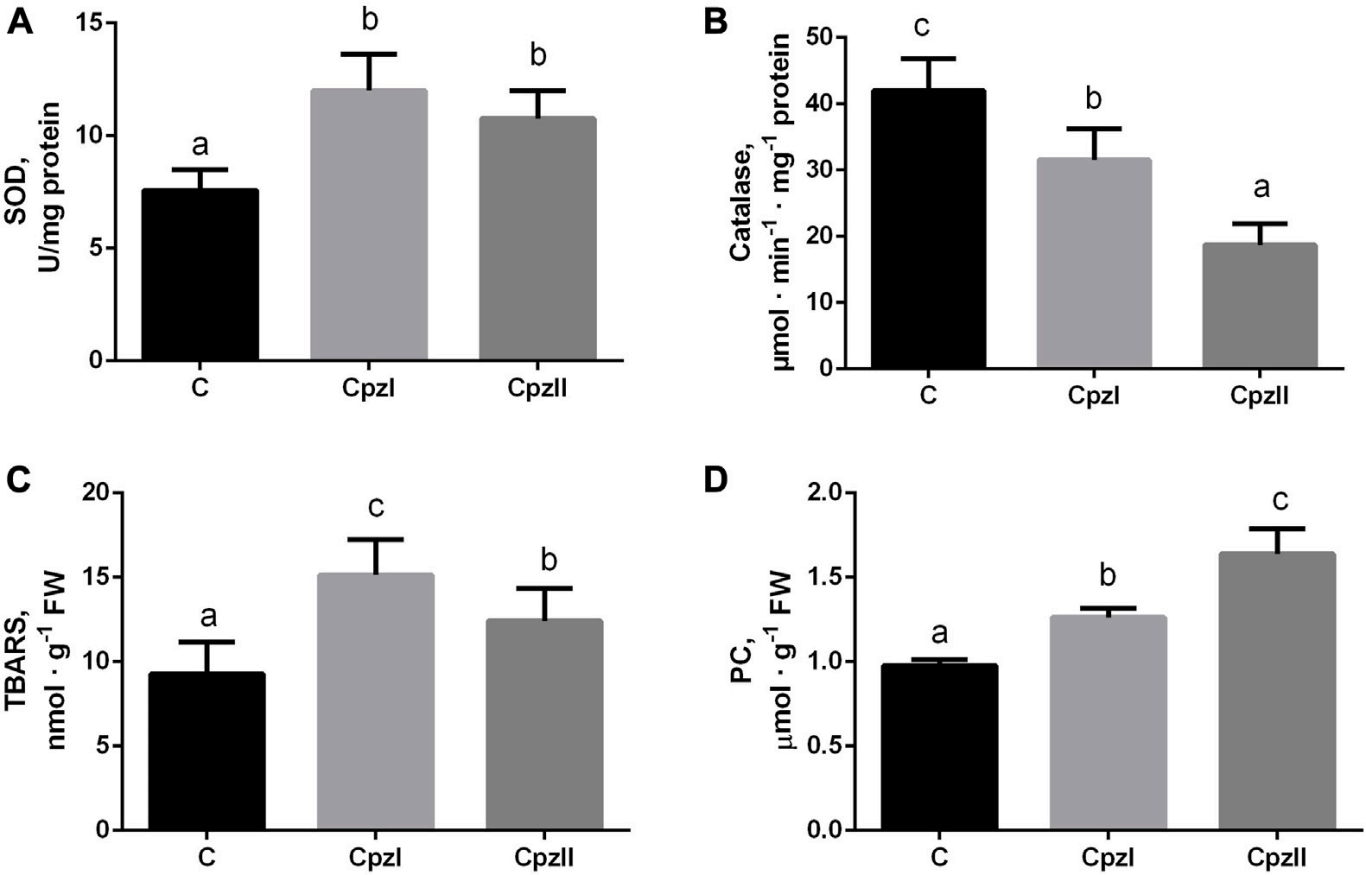
The antioxidant enzyme activities and manifestations of oxidative lesions in the digestive gland of bivalve molluscs *Mytilus galloprovincialis* following exposure to two concentrations of chlorpromazine (Cpz) for 14 days: **(A)**, superoxide dismutase; **(B)**, catalase; **(C)**, TBARS; **(D)**, Protein carbonyls. Groups: C - control group, Cpz 1–12 ng L^-1^of Cpz; Cpz 2–12 μg L^-1^of Cpz. Different letters above the columns indicate significant differences between groups, *M±SD, N = 8, p* < 0.05. Data were analyzed by using SPSS Statistics for Windows, Version 24.

The evaluation of the antioxidant manifestations detected opposite trends for each studied enzyme in both exposed groups (Figures 2A, B). The SOD activity increased, and catalase, particularly in Cpz 2 group, - decreased. This misbalance in the antioxidant activities was accompanied by the elevation of TBARS and protein carbonyls levels (Figures 2C, D).

#### 3.3.1 Exposed versus control

**FIGURE 3:**
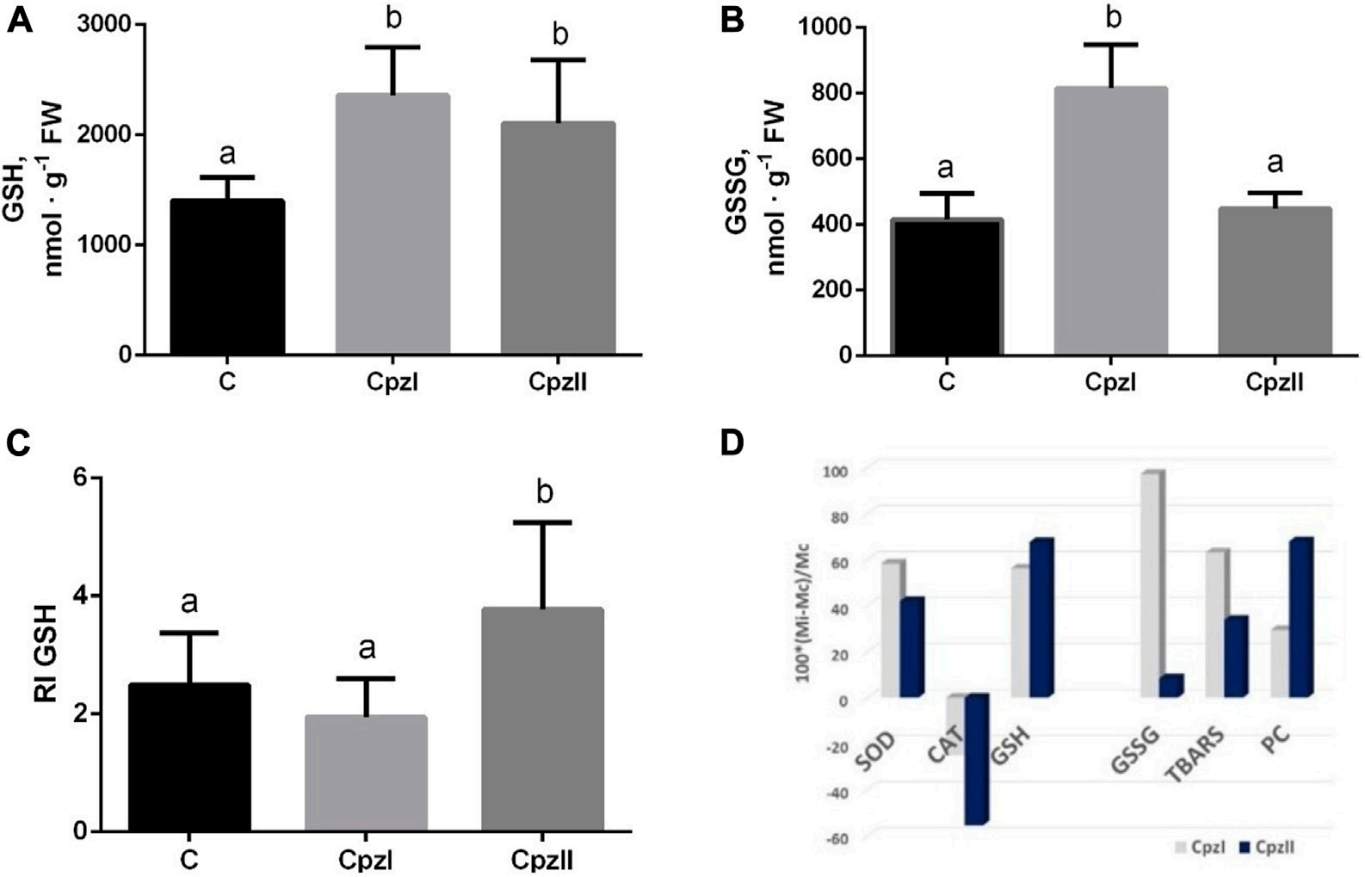
The concentrations of GSH **(A)**, GSSG **(B)**, redox index of glutathione **(C)**, and relative changes of each analyzed oxidative stress index **(D)** in the digestive gland of bivalve molluscs *Mytilus galloprovincialis* following exposure to two concentrations of chlorpromazine (Cpz) for 14 days. Groups: C - control group, Cpz 1–12 ng L^-1^of Cpz; Cpz 2–12 μg L^-1^of Cpz. Different letters above the columns indicate significant differences between groups, *M±SD, N = 8, p* < 0.05. Data were analyzed by using SPSS Statistics for Windows, Version 24.

The concentration of GSH increased similarly in both exposures to Cpz, whereas the level of GSSG increased only by the low Cpz concentration, and, consequently, RI GSH increased compared to control under the effect of CpzII only (Figures 3A–C).

The mean value of antioxidant/pro-oxidant manifestations (APB) in the control group was taken as 1.0. The calculations of its shift in the exposures indicated that in both exposed groups, despite the individual indexes had different concentration-dependent deviations (Figure 3D), APB decreased against control almost equally (by 52.8% and 50.8% in the Cpz 1 and Cpz 2 groups correspondingly).

## 4 Discussion

To obtain a complete understanding of the health status of the model organism, we conducted cell tests on both haemolymph and digestive gland cells. With regard to haemolymphatic cells, they represent the first line of defence against pathogens and xenobiotics and can be considered suitable biomarkers for monitoring the effects of environmental stress (Multisanti et al., 2023). Hemocytes can recognize and eliminate pathogens and xenobiotics through phagocytosis and the secretion of antimicrobial peptides, various humoral factors, and reactive oxygen intermediates (Impellitteri et al., 2022). By evaluating the viability of hemocytes, we gained valuable insights into the overall health status of our model organism. The viability of hemocytes is indicative of their capacity to respond to stressors and maintain their functional integrity in the presence of environmental challenges. The digestive gland (DG) of *M. galloprovincialis* assumes a crucial function in digesting nutrients vital for the organism’s survival. Moreover, the DG serves as a critical detoxification centre. Nevertheless, owing to its vulnerability to the buildup of pollutants, this organ can become a central target for environmental stressors. By assessing the viability of haemocytes together with that of digestive gland cells, we can understand how Cpz may influence the body’s immune defence and detoxification capabilities. NR uptake is a valuable indicator for assessing the stability of lysosomal membranes in bivalve cells. Lysosomes play a crucial role as cytoplasmic organelles, harbouring several hydrolytic enzymes with optimal activity at acidic pH levels. Their ability to degrade a wide range of biological molecules is crucial for cell health. It is particularly noteworthy that lysosomes act as specific targets for the toxic effects of many contaminants, accumulating pollutants and becoming sensitive indicators of cellular health. In mussels, the lysosomal vacuolar system is particularly important (Moore et al., 2004). Digestive gland cells, enriched with lysosomes, are a key interface between the organism and its environment, facilitating intracellular digestion. In addition, the small granulocytes of the haemolymph, also rich in lysosomes, contribute to digestion processes and innate immune response mechanisms (Martínez-Gómez et al., 2015). Efficient regulation of this process could contribute significantly to the ability of some organisms to adapt and survive in stressful and polluted habitats.

The trypan blue exclusion test allowed us to ascertain the number of viable cells in a cell suspension. In our results, only digestive gland cells showed substantial significance (*p* ≤ 0.01) in the Cpz 2 group compared to the control group, in both the NR and TB tests. The observed decline in cell viability suggests potential damage to the cell membrane. These findings align with similar studies reported in the literature (e.g., Pagano et al., 2022; Impellitteri et al., 2023a; Tresnakova et al., 2023a; Impellitteri et al., 2023b; Tresnakova et al., 2023b).

However, it is crucial to note that although the integrity of the cell membrane may remain intact, such as in haemolymph cells, the overall viability of the cell, including its growth and functionality, may still be affected.

While our viability tests indicated potential detrimental effects of Cpz on DG cells, the examination of these cells’ ability to regain their original volume when exposed to a hypotonic solution, as demonstrated by the RVD assay, did not reveal statistically significant differences among the treatment groups compared to the control group. Cells of osmoconform organisms can sense and respond to osmotic changes in their environment by adjusting volume (Tresnakova et al., 2023a; Impellitteri et al., 2023b; Tresnakova et al., 2023b). The RVD evaluation can detect changes in this process caused by pollutants, offering an accurate approach to evaluating cellular harm. In the Cpz 1 group, we noted a pattern in which DG cells seemed to struggle to revert to their original volume, leading to a prolonged state of swelling. Conversely, DG cells exposed to the highest Cpz concentration displayed atypical shrinkage patterns. It is crucial to emphasize that while these observations may appear notable in the graphical representation (Figure 1), they did not reach statistical significance in our analysis. We acknowledge that the absence of statistical significance indicates that these differences could be attributed to chance variation rather than true effects of Cpz exposure.

The response of oxidative stress is a common biological phenomenon that is caused by external or internal adverse effects in organisms, including bivalve molluscs (Fabbri and Franzellitti, 2016; Moreira et al., 2016; Paital et al., 2016; Gnatyshyna et al., 2020; Matozzo et al., 2020). However, the manifestations of oxidative stress differ depending on the severity of the impact (Lushchak, 2011; Moreira et al., 2016). In the present study, despite it was indicated the increase in both antioxidative (SOD, GSH) and oxidative (TBARS, PC, GSSG) manifestations (Figure 3D), the general direction of their imbalance was pro-oxidative. The catalase activity was the most vulnerable target of the pharmaceutical Cpz. Its decrease in both exposures seems to be the crucial event in the prooxidative shift that allows transient accumulation of hydrogen peroxide H_2_O_2_ and hydroxyl radical OH**·**, the reactive intermediates of the activity of SOD, resulting in greater oxidative lesions (Ransy et al., 2020; Alam et al., 2022). This manifestation is consistent with other results concerning the impaired antioxidant capacity in bivalve molluscs. For example, in the 15-day exposure of *M. galloprovincialis* to mixtures of non-steroidal inflammatory drugs ibuprofen and diclofenac and selective serotonin reuptake inhibitor fluoxetine without or along with the addition of copper, a catalase gene expression was downregulated. In the cladoceran species *Daphnia* magna, exposure to 1.0 µg of Cpz L^-1^ also caused a decrease in catalase activity, as the most prominent sign of oxidative stress (Oliveira et al., 2015).

Among indexes of oxidative stress, GSSG has shown the most selective concentration-depending response. The balanced increase of GSH and GSSG levels in the Cpz 1 group can be explained by the activation of glutathione peroxidase-related way of the destruction of H_2_O_2_ coupled with the oxidation of the GSH (Manduzio et al., 2005). Under exposure to higher Cpz concentration, the utilization of GSH seems to be reduced, which, in turn, leads to the elevated redox balance modulating cellular metabolism (Xiao and Loscalzo, 2020; Martyniuk et al., 2022b).

Importantly, the indicated features of the oxidative changes in the present study are similar to that reported early for the freshwater bivalve mollusc *Unio tumidus* subjected to Cpz in the concentration of 18.0 μg L^-1^ for 14 days (Khoma et al., 2020; Khoma et al., 2022). In particular, there were confirmed the responses of catalase, TBARS and GSH, and the total balance of antioxidants versus prooxidative changes (APB).

Phenothiazine and its derivatives including Cpz have been known for many years as potent antioxidants, based on its reducing activity via the formation of radical cation, in the interaction, for example, with the thiyl radical derived from GSH (Tamba and O`Neill, 1991). Nevertheless, the studies on vertebrates inform about its antioxidant or prooxidant effects. In fish *Carassius auratus*, acute toxicity of Cpz indicated as the median lethal concentration (LC_50_) in 24, 48 and 96 has 1.11, 0.43 and 0.32 mg L^-1^ correspondingly, was accompanied with the oxidative manifestations different depending on the time of exposure (Li et al., 2008).

Some signs of Cpz pro-oxidative effects were confirmed in the erythrocytes from human blood treated with Cpz in concentrations observed *in vivo* at therapeutic doses (1–100 µM) for 2 hours (Ficarra et al., 2016). However, unlike our finding for molluscs, these authors had shown almost twice reduce in GSH/GSSG ratio. In the brain of rats, the chronic intraperitoneal administration of Cpz (5 and 10 mg kg^-1^) induced the antioxidant enzyme SOD, but did not affect catalase, and inhibited lipid peroxidation (Roy et al., 1984).

Hence, our results confirm that the impact on the antioxidant-pro-oxidant balance is the intrinsic feature of Cpz toxicity. According to the intensity-based classifications of oxidative stress strong oxidative stress (Lushchak and Storey, 2021), or “oxidative distress” (Sies, 2017) was caused even by low pM and nM concentrations of Cpz, which induced the increase of the level of ROS-modified molecules and disturbed activities of antioxidant enzymes, and consequently, it has been implicated in the cellular injury.

Bivalve molluscs represent vulnerable to the oxidative impact of Cpz model organisms. The deleterious physiological effects of Cpz detected in the present study, obviously are the consequences of the biochemical changes caused by Cpz. Therefore, aiming to create a realistic view of the Cpz ecotoxicity on aquatic habitats it is crucially important to study its effect in prolonged exposures with a focus on the antioxidant capacity as the most probable target of this reductive active compound.

## 5 Conclusion

Based on our findings, it has been observed that Cpz (Chlorpromazine), even at low nanomolar concentrations, induces non-specific symptoms of biochemical and physiological disturbances in *M. galloprovincialis*, a species of marine mussel. These disturbances manifest in various ways and appear to affect the vitality of cells, leading to an imbalance in oxidative stress with a pro-oxidative shift. The impact of Cpz in *M. galloprovincialis* suggests that this substance can have detrimental effects on the mussels even at very low concentrations. In light of our findings, it is essential to consider the implications of potential environmental exposure to Cpz, as marine organisms such as mussels often come into contact with diverse pollutants and pharmaceuticals that enter aquatic environments. Further research and monitoring may be necessary to better understand the extent of Cpz’s effects on marine ecosystems and to assess potential risks to aquatic organisms like *M. galloprovincialis*. Understanding these impacts can aid in developing appropriate measures to protect aquatic environments and the organisms living within them.

## Data Availability

The original contributions presented in the study are included in the article/Supplementary Material, further inquiries can be directed to the corresponding author.
